# Remote intracranial recurrence of *IDH* mutant gliomas is associated with *TP53* mutations and an 8q gain

**DOI:** 10.18632/oncotarget.20951

**Published:** 2017-09-15

**Authors:** Shunsuke Nakae, Takema Kato, Kazuhiro Murayama, Hikaru Sasaki, Masato Abe, Masanobu Kumon, Tadashi Kumai, Kei Yamashiro, Joji Inamasu, Mitsuhiro Hasegawa, Hiroki Kurahashi, Yuichi Hirose

**Affiliations:** ^1^ Department of Neurosurgery, Fujita Health University, Toyoake, Japan; ^2^ Division of Molecular Genetics, Institute for Comprehensive Medical Science, Fujita Health University, Toyoake, Japan; ^3^ Department of Radiology, Fujita Health University, Toyoake, Japan; ^4^ Department of Neurosurgery, Keio University, Tokyo, Japan; ^5^ Department of Pathology, Fujita Health University, Toyoake, Japan

**Keywords:** IDH mutant gliomas, radiological classification for recurrence, Intracranial remote recurrence, TP53 mutations, 8q gain

## Abstract

Most *IDH* mutant gliomas harbor either 1p/19q co-deletions or *TP53* mutation; 1p/19q co-deleted tumors have significantly better prognoses than tumors harboring *TP53* mutations. To investigate the clinical factors that contribute to differences in tumor progression of *IDH* mutant gliomas, we classified recurrent tumor patterns based on MRI and correlated these patterns with their genomic characterization. Accordingly, in *IDH* mutant gliomas (*N* = 66), 1p/19 co-deleted gliomas only recurred locally, whereas *TP53* mutant gliomas recurred both locally and in remote intracranial regions. In addition, diffuse tensor imaging suggested that remote intracranial recurrence in the astrocytomas, IDH-mutant with *TP53* mutations may occur along major fiber bundles. Remotely recurrent tumors resulted in a higher mortality and significantly harbored an 8q gain; astrocytomas with an 8q gain resulted in significantly shorter overall survival than those without an 8q gain. OncoScan^®^ arrays and next-generation sequencing revealed specific 8q regions (i.e., between 8q22 and 8q24) show a high copy number. In conclusion, only tumors with *TP53* mutations showed patterns of remote recurrence in *IDH* mutant gliomas. Furthermore, an 8q gain was significantly associated with remote intracranial recurrence and can be considered a poor prognostic factor in astrocytomas, IDH-mutant.

## INTRODUCTION

Glioma is one of the most common brain tumors and has been classified according to its histological appearance. However, genetic and chromosomal markers that are strongly associated with patient prognosis such as *isocitrate dehydrogenase* (*IDH*) *1/2*, *TERT* promoter, 1p/19 co-deletions, *TP53*, and *ATRX*, have been reported, and their validity has been shown previously [1-11]. Based on these studies, most adult supratentorial gliomas can be genetically classified into *IDH* wild-type gliomas, *IDH* mutant gliomas with *TP53* or *ATRX* mutations, or *IDH* mutant gliomas with 1p/19q co-deletions [6, 8]. In the revised WHO classification, an *IDH* mutation and 1p/19q co-deletions are crucial for a diagnosis of adult supratentorial glioma. WHO grade II and III oligodendrogliomas harbor an *IDH* mutation and 1p/19q co-deletions [12]. Therefore, *IDH* and *TP53* (or *ATRX*) mutant gliomas are categorized into diffuse (WHO grade II) or anaplastic (WHO grade III) astrocytomas, IDH-mutant in the revised WHO classification.

In *IDH* mutant gliomas, it is well known that 1p/19q co-deleted gliomas (oligodendrogliomas in the revised WHO classification) have better prognoses; our previous study revealed that *IDH* mutant gliomas with wild-type *TP53*, which are almost identical to 1p/19q co-deleted gliomas, showed significantly better prognoses than *IDH* and *TP53* mutant gliomas (astrocytomas in the revised WHO classification) [8]. Recently, studies of recurrent *IDH* mutant gliomas have been increasing. We previously reported that copy number aberrations (CNAs) +8q, −9p, −11p, and +12p were frequently detected in recurrent tumor samples, suggesting that these chromosomal regions contain key molecules for rapid tumor progression in *IDH* mutant gliomas [8]. Another study of recurrent *IDH* mutant gliomas showed that the myelocytomatosis (MYC) signaling pathway is associated with tumor progression [13]. However, the differences in radiological recurrent patterns between these two subtypes are currently unknown.

To better understand the high recurrent subtype, (i.e., the *TP53* mutant group in *IDH* mutant gliomas), we investigated the differences between *TP53* mutant and 1p/19q co-deleted gliomas, using MRI-based recurrent pattern. We subsequently investigated correlations between recurrent patterns in *TP53* mutant gliomas and CNAs using metaphase comparative genomic hybridization (CGH), OncoScan^®^ array, and next-generation sequencing (NGS). Our investigations provide new insights into radiological recurrent patterns and their association with genetic information in diffuse and anaplastic astrocytomas, IDH-mutant.

## RESULTS

### Investigation of patterns in recurrent *IDH* mutant gliomas

**Figure 1:**
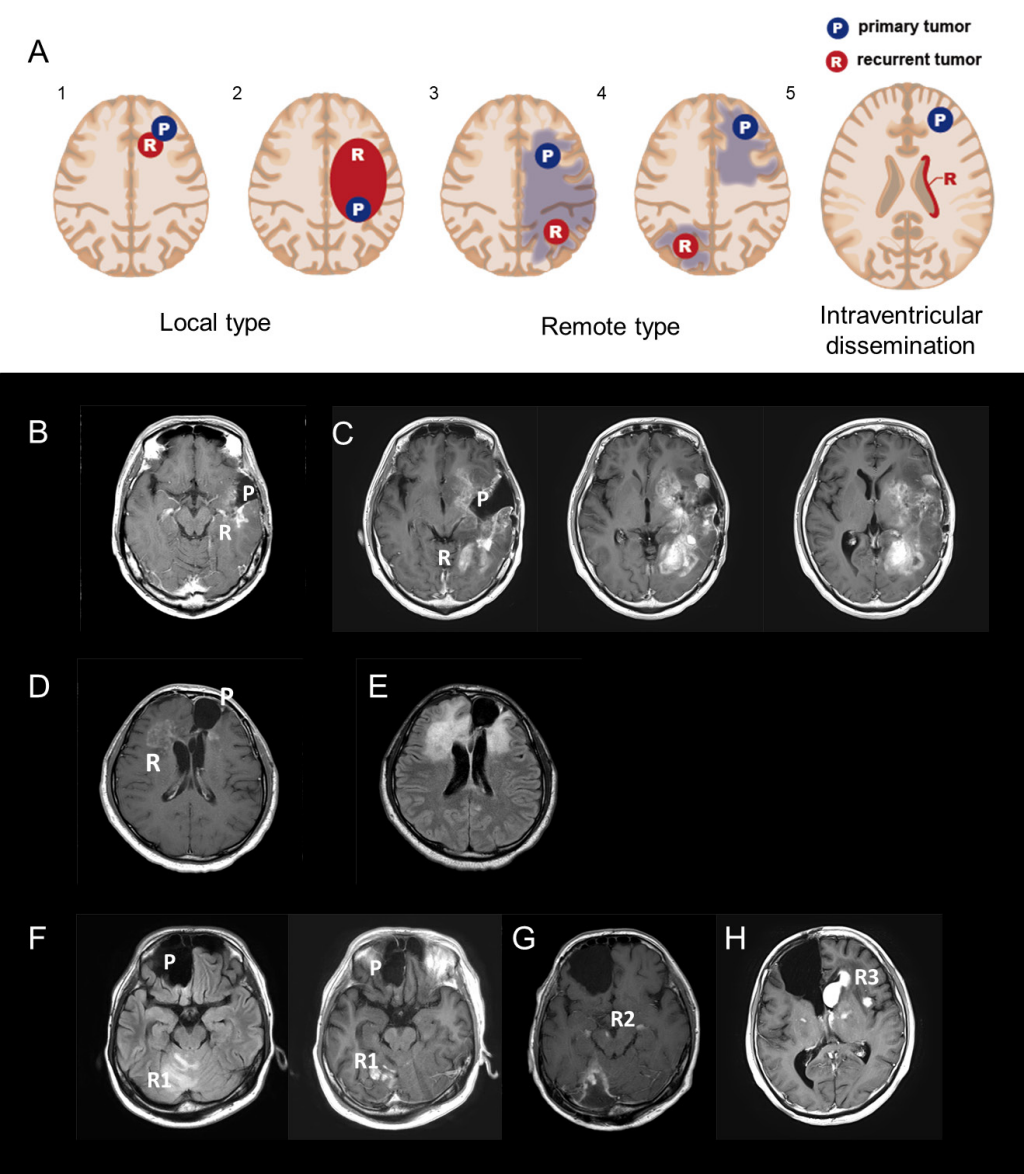
**A.** Five radiological recurrent patterns of gliomas: Type 1: local recurrence at the site of the primary lesion inside a single lobe; Type 2: diffuse enlargement from a primary lesion into different lobes; Type 3: an independent mass located in a different lobe, cerebellum, or brainstem that is continuous with the primary lesion in a T2/FLAIR high-intensity area; Type 4: a completely independent mass in a different lobe, cerebellum, or brain stem apart from the original lesion; and Type 5: intraventricular dissemination. B and C. A representative case of recurrent 1p/19q co-deleted glioma of Types 1 and 2 (W5). An MRI image (Gd-T1) taken 90 months after the first surgery (B); a recurrent contrast-enhanced tumor located around the cavity of the resected original tumor. At the time, the recurrent tumor was categorized as Type 1. MRI images (Gd-T1) after 115 months show a recurrent enlarged tumor located within the frontal and occipital lobes (C). D and E. A representative case of a *TP53* mutant glioma of Type 3 (M14). A Gd-enhanced independent tumor was detected from a primary lesion (D). A FLAIR image reveals two lesions that are continuous with a high-intensity area (E). F-H. A representative case of a *TP53* mutant glioma of Type 4 (M8). MRI images (FLAIR and Gd-T1) taken 61 months after the first surgery (F); an independent recurrent tumor, located in the right cerebellum, was detected apart from the primary tumor located in the right frontal lobe. An MRI image (Gd-T1) after 95 months shows that the recurrent tumor metastasized to the midbrain and cerebral peduncles (G). An MRI image (Gd-T1) after 100 months reveals that the recurrent tumors metastasized bilaterally to the basal nuclei (H). Abbreviation: Gd gadolinium.

Sixty-six patients with *IDH* mutant gliomas were investigated in the present study. Based on the revised WHO classification, tumor samples from their first surgery were diagnosed as 22 diffuse astrocytomas, IDH-mutant, 9 anaplastic astrocytomas, IDH-mutant, 17 oligodendrogliomas, IDH-mutant and 1p/19q co-deleted, 12 anaplastic oligodendrogliomas, IDH-mutant and 1p/19q co-deleted, and 5 oligodendrogliomas, NOS, indicating that all *IDH* mutant gliomas enrolled in this study were categorized as lower grade gliomas. According to sequence and copy number analyses, *IDH* mutant cases were divided into wild-type *TP53* and 1p/19q co-deletions (29 patients), wild-type *TP53* and other CNAs or without copy number data (8 patients), and mutant *TP53* (29 patients) (Table 1). Of these genetic subgroups, the numbers of recurrent cases were 13 (44.8-%), 1 (14.3-%), and 17 (58.6-%) cases, respectively. Mortality was 13.8-%, 0-%, and 27.6-%, respectively. We subsequently classified each recurrent case into five radiological patterns, excluding a contraindicated case for MRI (Figure 1A). Table 1 shows the radiological recurrent pattern for each genetic subgroup. Interestingly, *IDH* mutant gliomas with 1p19q co-deletions showed only local recurrences, whereas *IDH* mutant gliomas with *TP53* mutations showed both local and remote recurrences (Table 1); the Fisher’s exact test revealed that differences between these two subtypes were significant (*p* = 0.001). Mortality in remotely recurrent tumors was significantly higher than that in locally recurrent *IDH* mutant gliomas (*p* = 0.04).

**Table 1:** Recurrent patterns for genetic subtypes.

Pattern of recurrence	Wild-type *TP53* and 1p/19q co-deletion (*N* = 29)	Wild-type *TP53* and other CNAs or no CGH data (*N* = 8)	Mutant *TP53* (*N* = 28)
1. local	7 (14.3 %)	1 (0 %)	5 (0 %)
2. local	6 (50.0 %)	0	2 (50.0 %)
3. remote	0	0	2 (50.0 %)
4. remote	0	0	7 (71.4 %)
5. dissemination	0	0	2 (50.0 %)

Numbers in parenthesis indicate the percent mortality. Out of 66 patients, 1 patient was omitted from this classification because of a contraindication for MRI. 2 cases of dissemination overlapped with cases of patterns l and 4, respectively.

### Investigation of remote recurrences of *IDH* and *TP53* mutant gliomas

**Figure 2:**
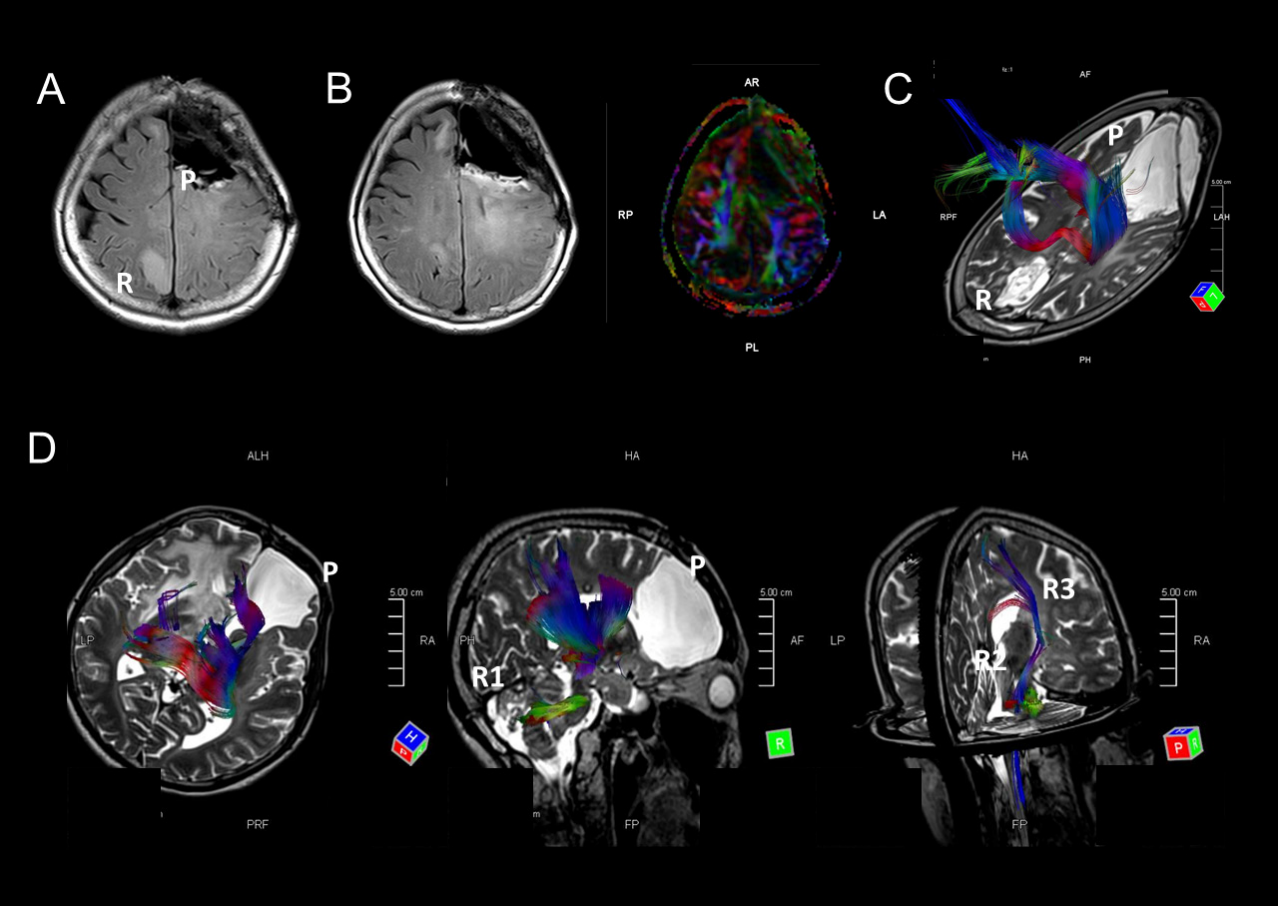
**A-C.** MRI images of case M1. A FLAIR image indicates that the tumor metastasized from the left frontal lobe to the opposite parietal lobe (A). FLAIR and DTI images at almost the same level indicate that the FLAIR high-intensity areas overlap with a major fiber bundle detected by DTI (B). A fiber tracking image indicates that fiber bundles connect the primary and recurrent tumor areas (C). D. Fiber tracking images show fiber bundles connect the primary tumor area to the right cerebellum *via* the right thalamus and brain stem. Other fiber bundles were detected from the brain stem to right basal nucleus. MRI images of this patient are shown in Figure 1F-1H.

Because *IDH* and *TP53* mutant gliomas (astrocytomas in the revised WHO classification) showed remote intracranial recurrences, we retrospectively investigated the primary and recurrent sites of the tumors. Six of nine cases were from a frontal lobe to the other frontal lobe, and one case (M1) was initially located in a region in the frontal lobe and recurred in contralateral parietal lobe (Figure 2A and B). In another case (M8), the primary tumor was located in the right frontal lobe, and the tumor recurred in the ipsilateral cerebellum, metastasized to the midbrain, and subsequently found bilaterally in the basal nuclei (Figure 1F-H). These findings led us to the hypothesis that remote intracranial recurrence in *IDH* and *TP53* mutant gliomas may occur via major fiber bundles. Since the corpus callosum interconnects the bilateral frontal lobes, we analyzed whether major fiber bundles lay between the primary and recurrent tumor sites using diffusion tensor imaging (DTI) for distantly recurrent cases (M1 and M8). Fiber tracking images showed that the major fiber bundles did connect the primary and recurrent areas in these two cases (Figure 2C and D). In addition, comparing images generated by fluid-attenuated inversion recovery (FLAIR) and DTI in M1, high-intensity regions in FLAIR images were consistent with the area of the fiber bundles (Figure 2B). These results suggest that the recurrence of *IDH* and *TP53* gliomas may occur via major fiber bundles.

In the other remotely recurrent case (M16), the tumor was located in the left frontal lobe, but then metastasized to the ipsilateral temporal lobe and cerebellum. However, these two recurrent tumors were located around the inferior horn of the lateral ventricle and fourth ventricle, in which ventricular dissemination was detected on MRI, suggesting that these recurrent tumors may move to other lobes via the cerebrospinal fluid rather than the major fiber bundles.

### Clinical features of diffuse and anaplastic astrocytomas, IDH-mutant with an 8q gain

**Figure 3:**
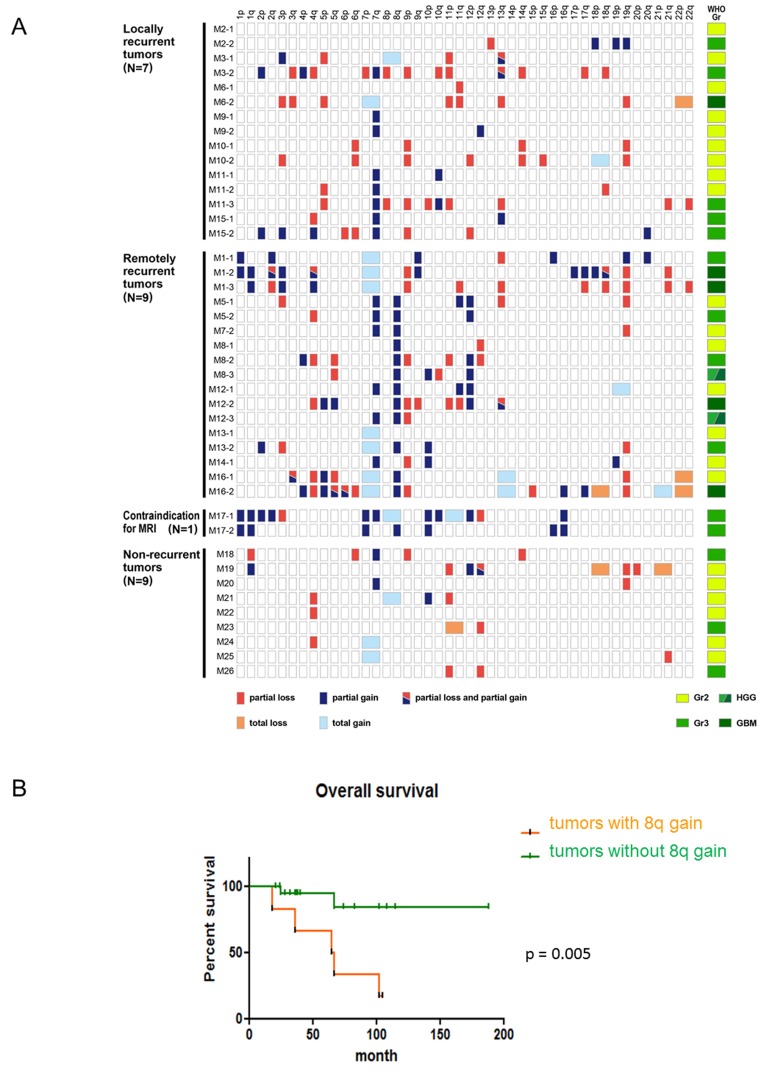
The clinical significance of an 8q gain in astrocytomas, IDH-mutant **A.** The copy number analysis obtained by metaphase comparative genomic hybridization (CGH) for *TP53* mutant gliomas reveal that a partial 8q gain was detected only in distantly recurrent tumors (*N* = 26). Red and blue boxes show partial chromosomal loss and gain, respectively. Light red and light blue boxes reveal total chromosomal loss and gain, respectively. A mixture of red and blue indicates that both partial gain and loss are detected in a single chromosomal arm. On the right side, the WHO grade of each sample is indicated. Light green, green, and deep green boxes show diffuse astrocytomas, IDH-mutant (grade II), anaplastic astrocytomas, IDH-mutant (grade III), and glioblastomas, IDH-mutant (grade IV) respectively. A mixture of green and deep green indicates tumor samples that were histologically diagnosed as high-grade gliomas. **B.** The Kaplan-Meier curve indicates that astrocytomas (*N* = 30; 26 cases harboring *TP53* mutations and 4 cases without *TP53* mutations) with an 8q gain demonstrate shorter survival than astrocytomas without an 8q gain.

As mentioned above, *IDH* and *TP53* mutant gliomas recurred in both remote and local regions. We investigated whether specific CNAs were associated with radiologically distinct patterns of recurrence. Interestingly, an 8q gain was frequently detected in patients with only a remote recurrence, but not in patients with a local recurrence (Table 2 and Figure 3A). The Fisher’s exact test revealed that this difference was statistically significant (*p* = 0.007). Although we performed this analysis for all chromosomal arms, no other CNAs demonstrated a significant difference between locally and distantly recurrent groups.

**Table 2a:** A list of patients who experienced recurrence of *IDH* mutant gliomas.

a. *IDH* mutant gliomas with 1p/19q co-deletions								
Case	Age, sex	WHO	Location	Recurrent pattern	PFS (mo)		OS (mo)	F/U (mo)
	CNAs							
W1-1	43M	O Gr3	Bi frontal	-	44	64		(dead)
	−1p, +1q12−32.1, −1q32.2-ter, +11, +17, +19p, −19q							
W1-2	49M	HGG	Bi frontal	1				
	−1p, −15q13-22.3, −19q							
W2-1	34M	O Gr2	Lt frontal	-	63	(alive)		65
	−1p, −14, −19q							
W2-2	39M	O Gr3	Lt frontal	1				
	−1p, −14q, −19q, +21q							
W3-1	53M	O Gr2	Lt frontal	-	104	(alive)		134
	−1p, +7, −15q, −19q, +22q							
W3-2	64M	O Gr3	Lt frontal ∼ parietal	2				
	−1p, +2, +7, −9p, +9q, −15q, −19q							
W4-1	35F	O Gr2	Rt frontal	-	52	(alive)		120
	FF−1p, −19q							
W4-2	40F	O Gr3	Rt frontal	1				
	NA							
W5-1	24M	O Gr2	Lt temporal	-	69	121		(dead)
	−1p, −19q							
W5-2	32M	O Gr3	Lt temporal ∼ insula	2				
	−1p, −4, +7q21.3-ter, +8, +11, −14q22-23, −18, −19q							
W5-3	33M	O Gr3	Lt temporal, insula, occipital	2				
	−1p, +3, −4, +5, +7, +9q, +10p, −10q, −13q, −15q11.2-22.3, +15q22.2-ter, −19q							
W5-4	34M	O Gr3	Lt temporal, insula, frontal, occipital	2				
	−1p, −4, −10q, −13q, −14q, −15qcen-21, +15q24-ter, −19q							
W6-1	40F	O Gr3	Lt frontal	-	105	(alive)		133
	−1p, +1q, +3, −9, +12q14, −15q, +17, +18, −19q, +20							
W6-2	49F	not clear	Lt frontal	1				
	NA							
W7-1	57M	O Gr2	Lt temporal	-	33	87		(dead)
	−1p, −14q13-24, −19q							
W7-2	63M	O Gr3	Lt temporal ∼ parietal	2				
	−1p, +7, −14q21-24.3, −15q15-22.1, −19q							
W7-3	64M	O Gr3	Lt temporal ∼ parietal	2				
	−1p, +7, −14q21-24.3, −15q15-22.1, −19q							
W8-1	41M	A Gr3	Lt frontal	-	3	76		(dead)
	NA							
W8-2	45M	O Gr3	Lt frontal, temporal, rt frontal	2				
	−1p, +7q, +8, +18p, −18q, −19q, +22q							
W9-1	29M	O Gr3	Lt frontal	-	49	(alive)		88
	−1p, −19q							
W9-2	34M	O Gr3	Lt frontal	1				
	−1p, −19q							
W10-1	33M	OA Gr2	Lt temporal	-	25	(alive)		52
	−1p, +2p, −9p, −19q							
W10-2	36M	O Gr3	Lt temporal	1				
	−1p, −17p, −18q, −19q							
W10-3	38M	O Gr2	Lt temporal ∼ parietal	2				
	NA							
W11-1	36F	O Gr3	Lt frontal	-	17	(alive)		49
	−1p, −19q							
W11-2	37F	O Gr3	Lt frontal	1				
	−1p, +1q, −2, +6, +7, +8, −9, +11, −16, +17, −18, +19p, −19q, +21, −22							
W12-1	38F	O Gr2	Lt frontal	-	16	(alive)		32
	−1p, −19q							
W12-2	39F		Lt frontal ∼ rt frontal	2				
	NA							
W13-1	44M	O Gr3	Lt frontal	-	15	(alive)		29
	−1p, −19q							
W13-2	45M		Lt frontal	1				
	NA							

An 8q gain was detected in 13 tumors harboring *IDH* and *TP53* mutations from 7 patients; tumors with an 8q gain consisted of 5 diffuse astrocytomas, 4 anaplastic astrocytomas, and 2 glioblastomas, IDH-mutant, whereas tumors without an 8q gain consisted of 17 diffuse astrocytomas, 10 anaplastic astrocytomas, and 3 glioblastomas, IDH-mutant (Table 2 and Figure 3A). The difference in WHO grades between tumors with and without an 8q gain was not significant. In 4 of 7 patients, tumor specimens from the first surgery harbored an 8q gain. In 2 patients, tumors from the second surgery harbored an 8q gain, although those from the initial surgery did not. In another patient, the tumor sample from the first surgery was not available, but the one from the second surgery harbored an 8q gain (Table 2 and Figure 3A). In all patients, the detection of an 8q gain preceded a remote recurrence: 43 (M5), 61 (M8), 24 (M12), 11 (M13), and 20 (M16) months before remote recurrence, suggesting that an 8q gain could be a driver of such a recurrence.

Tables 1 and 2 reveal that remote recurrences of *IDH* and *TP53* mutant gliomas result in poor prognoses; therefore, we hypothesized that an 8q gain may be a poor prognostic factor in *IDH* and *TP53* mutant gliomas. Because the revised WHO classification genetically defines astrocytomas, IDH-mutant as gliomas with *IDH* mutations and an intact 1p/19q, the overall survival curves were analyzed adding 3 *IDH* mutant gliomas with wild-type *TP53* and an intact 1p/19q. Accordingly, Figure 3B suggests that astrocytomas, IDH-mutant without an 8q gain had significantly better prognoses than those harboring an 8q gain (N = 30; *p* = 0.005). Because all *IDH* mutant gliomas with an 8q gain harbor *TP53* mutations (Table 2 and Figure 3A), these results suggest that remote recurrence depends on a *TP53* mutation, is significantly associated with an 8q gain, and that the CNA contributes to a poor prognosis in diffuse and anaplastic astrocytomas, IDH-mutant.

### Clinical significance of a high number copy gain in 8q terminal regions

We first performed an OncoScan^®^ array for a paired set of primary and recurrent tumor samples to explore malignant or recurrent factors of *IDH* and *TP53* mutant gliomas. We performed this array on M6, M10, and M11 from a local type and on M5 and M8 from a remote type (Table 2b). Although this array provided us with much information, high copy number gain in 8q terminal regions was detected only in remotely recurrent tumors. Therefore, we focused on only high copy number gain regions and switched to copy number analysis using NGS, which also provided us information on the high copy number. We first conducted NGS for M5, M6, M8, M10, and M11, and compared these data from the OncoScan^®^ array with those from NGS. Analyzed data, including copy number loss, gain, and high copy number gain, from OncoScan^®^ and NGS were quite similar, although the array could detect much shorter CNAs than could NGS.

**Table 2b:** A list of patients who experienced recurrence of *IDH* mutant gliomas.

b. *IDH* mutant gliomas with *TP53* mutation							
Case	Age, sex	WHO	Location	Recurrent pattern	sPFS (mo)	OS (mo)	F/U (mo)
	*TP53* mutation		CNAs				
M1-1	48F	A Gr3	Lt frontal	-	177	(alive)	183
	R175H (exon 5)		+1pter-34.2, +2q36-ter, +7, +9q34.1-ter, −13q14.3-21.3, +16pter-12, +19qcen-13.3, −21q				
M1-2	63F	GBM	Lt frontal	1			
			+1p, +1q, −2q24.3-31, +2q33-ter, +3pter-23, −4q21.3-ter, +4qcen-13.1, +7, −9p, +9q21.2-21.3, −10q, −13q14.1-22, +17p13-cen, +17qcen-21.2, +18p11.2-cen, +18qcen-21.1, −18q22-ter, −19q, −21q,				
M1-3	63F	GBM	Rt parietal	4			
			+1q, −2q24.3-32.1, +3p, +4qcen-13.1, −4q21.1-ter, +7, −9p, −11q, −13q14.2-22, −17q25-ter, −18q21.3-ter, −19q, −21q, −22q				
M2-1	22F	A Gr2	Rt insula	-	72	(alive)	115
	H193Y (exon 6)		none				
M2-2	28F	A Gr3	Rt insula, temporal, frontal	2			
			−13q22, +18p, +19, −X				
M3-1	46F	A Gr2	Lt frontal	-	26	(alive)	37
	R306* (exon 8)		+3p, −5p, +8 −11p, −13q12.1-21.3, +13q22-ter				
M3-2	49F	A Gr3	Lt frontal	1			
			+2p, −3q21-ter, +4pter-13.1, −4q21.1-ter, −7p, +7q, −8pter-23.1, −9p, −10q, −11p, −13qcen-22, +13q32-ter, −14q, −17q, −18q				
M3-D	50F	No OP	Ventricular dissemnaiton	5			
M4-1	34M	A Gr3	Lt frontal	NA	10	14	(dead)
	IHC						
M4-R	35M	No OP	Rt frontal	4			
M5-1	44F	A Gr2	Rt frontal	-	27	65	(dead)
	R273H (exon 8)		−3p22-21, +7q, +8q22-ter, +11q23.3-ter, +12p, −13q21-31, −19q				
M5-2	46F	A Gr3	Rt frontal	1			
			−4q28-ter, +7q, +8q23-ter, +12p, −Xq				
M5-R	47F	No OP	Lt frontal	4			
M6-1	22F	A Gr2	Lt frontal	-	78	(alive)	108
	R248W (exon 7)		−11q22-23.1				
M6-2	29F	GBM	Lt frontal	1			
			−3pter-3q24, −5p, +7, −11p, −11q22-23.1, −13q, −19q, −22, −X				
M7-1	22M	A Gr2	Rt frontal	-	20	36	(dead)
	IHC		NA				
M7-2	23M	A Gr2	Rt frontal, Lt frontal	4			
			+7q, +8q, −19q				
M8-1	33M	A Gr2	Rt frontal	-	12	(alive)	100
	R273H (exon 8)		+8q22.3-ter, −12q13-24.1				
M8-2	34M	A Gr3	Rt frontal	1			
			+4p, −4q, −5qcen-13, −5q21-ter, +8q13-ter, −9pter-21.3, −11p, +12p, −12q22-23				
M8-3	38M	HGG	Rt cerebellum	4			
			−5q31.1-ter, +8q22.3-ter, +10p, −10q, +12p				
M8-R	41M	No OP	midbrain	4			
M8-R	42M	No OP	Bi basal nucleus	4			
M9-1	61F	A Gr2	Rt frontal	-	20	(alive)	74
	Y163C (exon 5)		+7q31-ter, −X				
M9-2	64F	A Gr2	Rt frontal	1			
			+7q31.1-ter, +12q22-ter, −X				
M10-1	30F	A Gr2	Rt frontal	-	41	(alive)	67
	D281G (exon 8)		−6q, −9p, −14q22-ter, −19q				
M10-2	35F	GBM	Rt frontal	1			
			−3p, −6q, −9p. −12p, −14q, −15q, +18, −19q				
M11-1	41M	A Gr2	Lt frontal	-	47	(alive)	78
	R175H (exon 5)		+7q, +10q24-ter				
M11-2	45M	A Gr2	Lt frontal	1			
			−5p, +7q, −18q21.1-ter				
M11-3	47M	A Gr3	Lt frontal	1			
			−5p, +7q, −8pter-23.1, −9p, −10pter-13, +10q26.1, −11p, −13qcen-22, −21q21, −22q, −X				
M12-1	26M	ND	Rt frontal	-	18	67	(dead)
	Y236D (exon 7)		+7q, +8q22.1-ter, +11q23.3-ter, +12p, +19				
M12-2	30M	GBM	Rt frontal, lt frontal	4			
			−4q28-ter, +5pter-q23.3, +7q, +8q, −9p, −9q, −11pter-15.1, −11q23.1-ter, +12p, −13q21.1-22, +13q31-ter				
M12-3	31M	HGG	Rt frotanl , lt frontal	4			
			+7q, +8q, −9p, −X				
M13-1	37F	A Gr2	Lt insula	-	29	102	(dead)
	Y220C (exon 6)		+7				
M13-2	44F	A Gr3	Lt insula, frontal	2			
			+2p, −3p21.3-11.2, +7, +8q23-ter, +10pter-12.3, −19q13.2-ter				
M13-3	45F	A Gr3	Rt frontal	4			
			NA				
M14-1	30M	A Gr2	Lt frontal	-	9	(alive)	19
	G287E (exon 8)		+7q, −9p, +10p, +19p				
M14-2	31M	A Gr3	Lt frontal	1			
			NA				
M14-R	32M	No OP	Rt frontal	3			
M15-1	48M	A Gr3	Lt frontal	-	51	67	(dead)
	R175H (exon5)		−4q13-21, +7q, +13q31-ter, +X				
M15-2	52M	A Gr3	Lt frontal ∼ parietal	2			
			+2pter-22, +3pter-23, +4q21-24, +4q26-33, −6pter-22, −6q21-ter, +7q, −9pter-21, −12p13, +20q				
M16-1	25M	A Gr2	Lt parietal	-	15	25	(dead)
	IHC		−4q22-ter, +5pter-23.3, −5q31.2-ter, +7, +8q, +13, −19q, −22				
M16-2	27M	GBM	Lt Parietal	1			
			−3q11.2-24, +3q24.1-ter, +4p, −4q, +5pter-5q23.3, −5q31.2-ter, −6pter-22.1, +6p22.2-18.3, −6q21-26, +7, +8q, −9p, +13, −14q, +16q, +17q, −18, −19q, +21, −22				
M16-RD	27M	No OP	Lt parietal, temporal, cerebellum	4			

This table includes age at diagnosis, gender, histology, tumor location, radiological recurrence pattern, PFS, OS, follow-up time in months, location of the TP53 mutation (TP53 mutant tumors only), and CNAs obtained by metaphase CGH for 1p/19q co-deleted gliomas and TP53 mutant gliomas. The letters ‘R’ and ‘D’ indicate that remote recurrence and intraventricular dissemination were detected, respectively, although additional surgery was not performed. Abbreviation: CNA copy number aberration, PFS progression-free survival, OS overall survival, F/U follow-up, A diffuse or anaplastic astrocytoma, IDH-mutant, O (anaplastic) oligodendroglioma and 1p/19q-codeleted, IDH-mutant, HGG high-grade glioma, Gr grade, IHC immunohistochemistry

**Figure 4:**
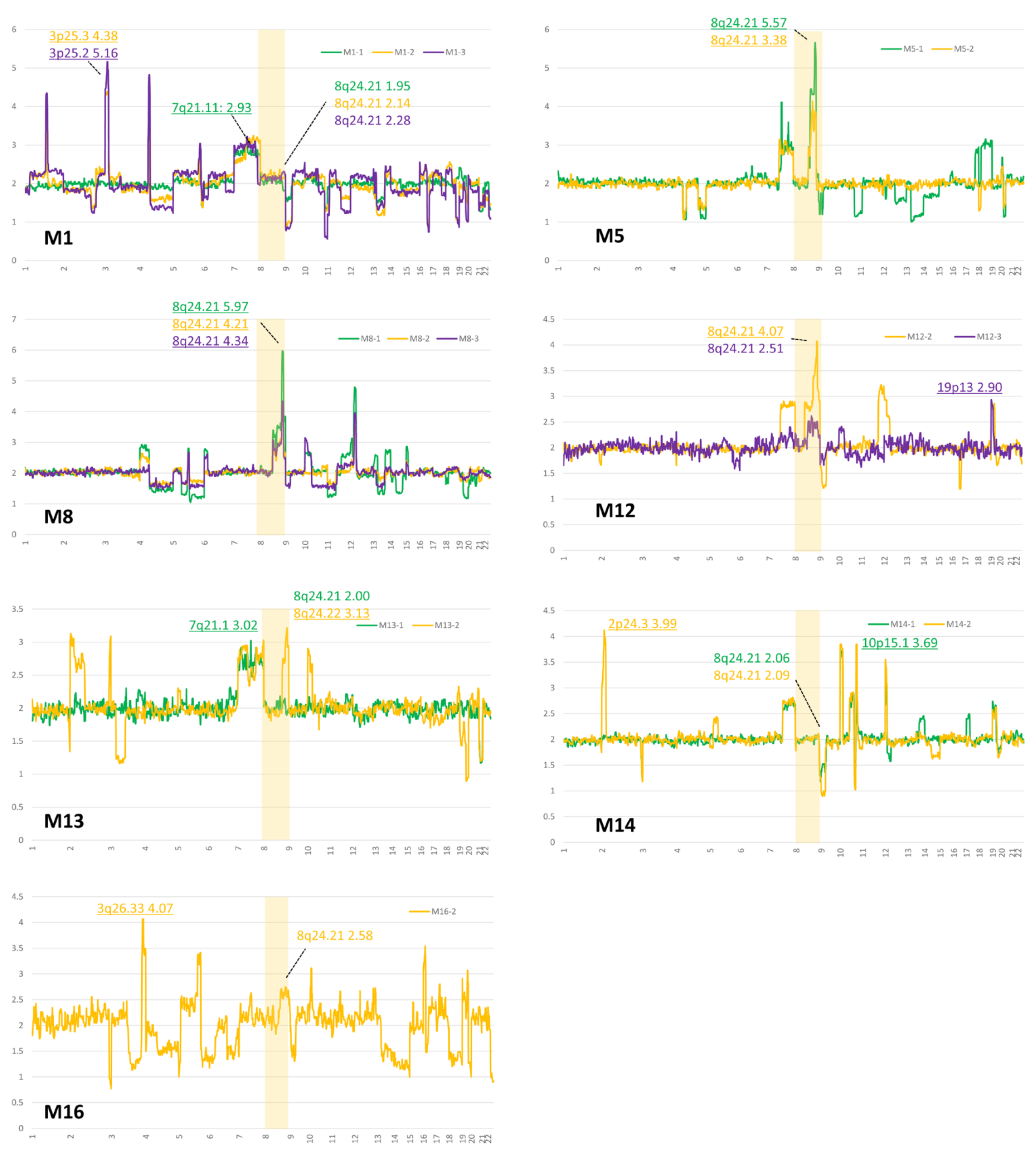
Calculated copy numbers obtained by NGS in paired samples from patients with astrocytomas with remote recurrences (M1, M5, M8, M12, M13, and M16) Green, yellow, and purple lines indicate the first, second, and third surgeries for patients, respectively. An area of chromosome 8 is highlighted, and the highest copy number of 8q is shown, because remotely recurrent *IDH* and *TP53* mutant gliomas are significantly associated with an 8q gain. The highest copy number is underlined in each sample.

We performed an NGS copy number analysis of all tumor samples from *IDH* and *TP53* mutant gliomas, obtaining reliable data from 28 samples (17 patients). Figure 4 revealed NGS-based copy number data from 7 patients who had remote tumor recurrence during their clinical course. Accordingly, specific regions of 8q between 8q23.3 and 8q24.23 showed the highest copy number in four patients (Figure 4). In M1 and M16, recurrent tumors had relatively high copy numbers of 8q: 2.31 and 2.75, respectively, suggesting that these tumors have short gains in the 8q region. Only one patient (M14) did not have a high copy number in 8q terminal regions in remotely recurrent tumors. No other patients in the *IDH* and *TP53* mutant group harbored an 8q gain. In distantly recurrent tumors, regions 2p25 to 2p24 (M13, M14, and M16), 10p15 to 10p14 (M8, M12, M13, M14, and M16), and 12p (M8, M12, M14, and M16) were frequently detected as high copy number chromosomal regions.

## DISCUSSION

In the present study, we focused on the recurrence of *IDH* mutant gliomas, particularly those harboring *TP53* mutations and found three major results: First, we clarified that only tumors with *TP53* mutations showed remote intracranial recurrence and that the recurrence might occur via major fiber bundles. Second, we reported that an 8q gain was significantly associated with distant recurrence and, according to a survival curve, can be used as a poor prognostic marker in astrocytomas, IDH-mutant. Third, NGS copy number analysis revealed that an 8q gain is a high copy number gain in many cases. Although the number of examined cases were relatively low, they all consisted of patients who were admitted to a single center (Fujita Health University); thus, these patients were followed continuously, enabling us to demonstrate novel radiological patterns in recurrent *IDH* mutant gliomas and their association with genetic information.

In *IDH* mutant gliomas, *TP53* mutant gliomas recurred in remote intracranial regions, whereas 1p/19q co-deleted gliomas only recurred locally, findings that have not been reported previously. A rare case of anaplastic astrocytoma, IDH-mutant that showed trans-tentorial metastasis was previously reported [14]. In this case, the location of the primary lesion was the right temporal lobe and the recurrent tumor was detected in the right cerebellum. Genetic analysis indicated that the tumor harbored an *IDH1* mutation but did not harbor the 1p/19q co-deletions, suggesting that this case was probably a *TP53* or *ATRX* mutant and consistent with our results. Because *TP53* mutant and 1p/19q co-deleted gliomas are thought to be astrocytic and oligodendroglial tumors, respectively, the difference in the radiological recurrent patterns may depend upon cell type. Moreover, specific molecular changes, such as an 8q gain, may provide the tumors with an ability to metastasize to a different lobe. 8q gains were detected only in *IDH* mutant gliomas harboring a *TP53* mutation, and remote recurrence occurred a few years after their initial detection (Table 2b). These results suggest that *TP53* mutations cause CNAs in specific chromosomal areas, such as 8q, and the high copy number increase in the 8q terminal region works as a driver for the remote recurrence of *IDH* mutant gliomas; thus, the key molecules for remote recurrence may be found in this chromosomal area.

We also revealed that an 8q gain is a poor prognostic factor in astrocytomas, IDH-mutant and the calculated copy number score in 8q, especially between 8q22 and 8q24, was high (Figures 3 and 4). Previous studies have focused on 8q, particularly 8q24 and *c-MYC*, which resides in the 8q24.21 region, in searches of human cancers, including gliomas. Kitange et al. reported that an 8q gain was significantly associated with shorter survival in cases of oligodendrogliomas and oligoastrocytomas [15]. Faria et al. reported that a *c-MYC* copy number gain analyzed by FISH was associated with c-MYC expression in astrocytic tumors [16]. Recently, Bai et al. reported that the MYC signaling pathway is strongly associated with tumor progression in *IDH1* mutant gliomas [13]. Previous studies have reported that single nucleotide polymorphisms located in 8q24.21 were strongly associated with tumor development [17, 18]. Apart from gliomas, the chromosomal region 8q24.21 has been a region of interest in human cancers. Recently, long noncoding RNA transcripts, *CCAT2* and *PVT1* located on 8q24.21, were reported to be associated with MYC dysfunction [19-22]. Our study also indicated that a high copy number increase in 8q24.21 was associated with tumor progression and/or intracranial metastasis in astrocytoma defined by the revised WHO classification. Although our findings may provide novel insights into how gains of 8q terminal regions affect the prognoses of patients with diffuse and anaplastic astrocytomas, we need to explore how an 8q gain causes remote intracranial recurrence.

The present study has some limitations. First, although fiber tracking images were used to show that major fiber bundles were detected between the primary and recurrent tumors in remotely recurrent tumors, such information alone cannot be used as proof of tumor migration via the major fiber bundles. Previous work focused on tumor migration indicated that Rho-GTPase is associated with tumor migration and invasion in GBM cells [23]. However, the mechanisms of tumor migration in *IDH* and *TP53* mutant gliomas are unclear. Therefore, we will next focus on how a tumor migrates to a different lobe. Second, the quantity and quality of some samples were not high enough for laboratory work, particularly in biopsy cases or cases where only formalin-fixed paraffin-embedded (FFPE) specimens were available; thus, we could not analyze all tumor samples using the same methods. In some cases, we examined *TP53* mutation status using immunohistochemical staining for TP53 and ATRX (Table 2b). In addition, treatments for *IDH* and *TP53* mutant gliomas are not uniform: Some patients undergo chemotherapy as their initial adjuvant therapy and others undergo concurrent chemotherapy and radiotherapy. However, in recurrent cases of *IDH* and *TP53* mutant gliomas in this study, all patients underwent both radiotherapy and chemotherapy during their clinical course.

In conclusion, we provide evidence of the characteristics of astrocytomas harboring *IDH* and *TP53* mutations, including their radiological recurrent pattern, prognoses, and malignant factors. These findings may help to design therapeutic strategies for this genetic type of glioma, as current treatments are controversial [24]. However, it is unknown how the high copy number gain of 8q terminal regions promotes malignancy and/or remote intracranial recurrence; these questions should be clarified in the future.

## MATERIALS AND METHODS

### Patients and tumor samples

Since *IDH* wild-type and mutant gliomas show different characteristics, including genetic background, patient background, and radiological features [25, 26], we focused on only *IDH* mutant gliomas in the present study. We retrospectively analyzed 66 patients with *IDH* mutant gliomas that had been surgically resected at Fujita Health University between 2001 and 2016. MRI was preoperatively performed for glioma patients except for MRI contraindicated cases. After tumor resection, specimens were histologically diagnosed by a neuropathologist as (anaplastic) astrocytomas, (anaplastic) oligodendrogliomas, and glioblastomas. Resected glioma samples were preserved as frozen tissues and/or FFPE samples.

### MRI-based recurrent classification

Based on MRI, we classified tumor recurrence into the following five patterns: Type 1: local recurrence at the primary lesion inside a single lobe; Type 2: diffuse enlargement from the primary lesion to into a different lobe; Type 3: an independent mass located in a different lobe, cerebellum, or brainstem that is continuous with the primary lesion in a T2/ FLAIR high-intensity area; Type 4: a completely independent mass in a different lobe, cerebellum, or brain stem apart from the original lesion; and Type 5: intraventricular dissemination (Figure 1A). An independent mass was evaluated using the following sequences: T1-Gd, if it was contrast-enhanced; T2; and/or FLAIR. Intraventricular dissemination was radiologically defined if contrast-enhancement on MRI was detected in the ventricles. We defined Types 1 and 2 as a local type, and Types 3 and 4 as a remote type. Intraventricular dissemination can be complicated by the other types. Representative cases for Types 2, 3, and 4 are shown in Figure 1B and C, 1D and E, and 1F-H, respectively.

We utilized DTI for surgical preparation in cases where tumors were located close to important long neural fibers. We analyzed DTI images using a fiber tracking method to examine correlations between tumor locations and major fiber bundles. We set up regions of interest (ROIs) between primary and recurrent lesions, allowing us to visualize the major fiber bundles within the ROIs.

### DNA preparation and mutation analyses

Genetic analysis of resected gliomas was approved by the Ethics Committee of Fujita Health University (Approval number: 11-106). Written informed consent was obtained from each patient. DNA was extracted from freshly frozen tissue using DNeasy blood and tissue kits (QIAGEN, Netherlands), and DNA from FFPE samples was extracted using DNA FFPE tissue kits (QIAGEN) or REPLI-g kits (QIAGEN). DNA was quantified using absorptiometric methods. Mutation analyses of *IDH1*/*2* and *TP53* mutations were conducted using Sanger’s method, as previously described [8]. Because previous studies showed that most missense mutations were found in exon 5 to 8 [27-29], we investigated *TP53* mutations in these areas. Primers for each gene and exon were selected from those described in previous studies [29-32]. In some cases, including biopsy cases, we could not obtain a sufficient quantity of DNA for the direct sequencing of *TP53*. In such cases, we substituted ATRX and TP53 immunohistochemistry for the *TP53* mutation analysis.

### Metaphase CGH

Using metaphase CGH, we detected CNAs whose chromosomal sizes were greater than 10 Mb. The metaphase CGH analysis was performed, as previously described [33]. Resected tumor tissues were removed from FFPE samples based on histological appearance, particularly MIB-1 density. Tumor DNA was amplified using degenerate oligonucleotide-primed PCR (DOP-PCR). Blood lymphocyte DNA from healthy donors was used as a control. DNA was labeled with biotin-deoxyuridine triphosphate (Roche, Switzerland) after amplification. The labeled DNA from tumors and normal tissues was subsequently hybridized to normal metaphase spreads. After unhybridized probes were washed away, the spreads were counterstained with 4,6-diamino-2-phenylindole, and the fluorescence intensity for each chromosome was evaluated using CytoVision software (Applied Imaging, MI, USA). This analysis was performed for 78 tumor samples from 58 patients.

### OncoScan^®^ FFPE assay

The OncoScan^®^ array (Affymetrix, CA, USA) was used to analyze fragmented genomic DNA (gDNA) from FFPE samples using molecular inversion probes. Eighty micrograms of gDNA from tumor samples were used for the analysis. Genomic probes were amplified by PCR via the process of annealing, gap-fill probe ligation, and linearization. After amplification, the amplicons were fragmented and hybridized to the arrays for 18 hours. Arrays were subsequently washed, stained, and loaded onto the fluidic station. Arrays were then scanned, and the files were analyzed by OncoScan^®^ Nexus Express software (Nexus). Overall genomic information for paired tumor samples was deposited in GEO; the accession number for our data is GSE94652 (http://www.ncbi.nlm.gov/geo/query/acc.cgi?acc=GSE94652). The analysis of array data was performed using 10 paired tumor sample from 5 patients in the *IDH* and *TP53* mutant tumor group.

### High copy number analysis by NGS

We performed comprehensive copy number analysis including high copy number gain from resected tumor samples using NGS, according to the manufacturer’s protocol (Illumina, CA, USA). Extracted DNA was amplified by whole genome amplification using SurePlex. One nanogram of each sample was tagged and fragmented, and then prepared for NGS analysis. After purification and normalization of the DNA libraries, the libraries were sequenced using the VeriSeq PGS Kit MiSeq. The sequencing data were analyzed using BlueFuse Multi Software. In this analysis, the mean copy number value was calculated for each 1-Mb window, with 2,500 windows covering the entire chromosomal region. We defined data as low quality if the score of the overall noise was greater than 0.3. We performed copy number analysis using NGS for patients in the *IDH* and *TP53* mutant group. Reliable data were obtained from 28 samples from 17 patients.

### Statistical analysis

Variables between two recurrent tumor types were analyzed by the Fisher’s exact test. Prognoses of patients were calculated according to their overall survival (OS). OS was defined as the date of first surgery until the date of death. Kaplan-Meier curves were generated, and Cox log-rank tests were used for group comparisons. *P* values less 0.05 were considered statistically significant.

## Abbreviations

IDH: isocitrate dehydrogenase
WHO: World Health Organization
MRI: magnetic resonance imaging
DTI: diffusion tensor imaging
GBM: glioblastoma multiforme
CGH: comparative genomic hybridization
NGS: next-generation sequencing
FFPE: formalin-fixed paraffin-embedded

## References

[1] Parsons DW, Jones S, Zhang X, Lin JC, Leary RJ, Angenendt P, Mankoo P, Carter H, Siu IM, Gallia GL, Olivi A, McLendon R, Rasheed BA (2008). An integrated genomic analysis of human glioblastoma multiforme. Science.

[2] Yan H, Parsons DW, Jin G, McLendon R, Rasheed BA, Yuan W, Kos I, Batinic-Haberle I, Jones S, Riggins GJ, Friedman H, Friedman A, Reardon D (2009). IDH1 and IDH2 mutations in gliomas. N Engl J Med.

[3] Killela PJ, Pirozzi CJ, Healy P, Reitman ZJ, Lipp E, Rasheed BA, Yang R, Diplas BH, Wang Z, Greer PK, Zhu H, Wang CY, Carpenter AB (2014). Mutation in IDH1, IDH2, and in the TERT promoter define clinically distinct subgroups of adult malignant gliomas. Oncotarget.

[4] Cairncross JG, Ueki K, Zlatescu MC, Lisle DK, Finkelstein DM, Hammond RR, Silver JS, Stark PC, Macdonald DR, Ino Y, Ramsay DA, Louis DN (1998). Specific genetic predictors of chemotherapeutic response and survival in patients with anaplastic oligodendrogliomas. J Natl Cancer Inst.

[5] Jiao Y, Killela PJ, Reitman ZJ, Rasheed AB, Heaphy CM, de Wilde RF, Rodriguez FJ, Rosemberg S, Oba-Shinjo SM, Nagahashi Marie SK, Bettegowda C, Agrawal N, Lipp E (2012). Frequent ATRX, CIC, FUBP1 and IDH1 mutations refine the classification of malignant gliomas. Oncotarget.

[6] Suzuki H, Aoki K, Chiba K, Sato Y, Shiozawa Y, Shiraishi Y, Shimamura T, Niida A, Motomura K, Ohka F, Yamamoto T, Takahashi K, Ranjit M (2015). Mutational landscape and clonal architecture in grade II and III gliomas. Nat Genet.

[7] Sabha N, Knobbe CB, Maganti M, Al Omar S, Bernstein M, Cairns R, Ҫako B, von Deimling A, Capper D, Mak TW, Kiehl TR, Carvalho P, Garrett E (2014). Analysis of IDH mutation, 1p/19q deletion, and PTEN loss delineates prognosis in clinical low-grade diffuse gliomas. Neuro Oncol.

[8] Nakae S, Sasaki H, Hayashi S, Hattori N, Kumon M, Nishiyama Y, Adachi K, Nagahisa S, Hayashi T, Inamasu J, Abe M, Hasegawa M, Hirose Y (2015). PCR-based simple subgrouping is validated for classification of gliomas and defines negative copy number aberrations in IDH mutant gliomas. PLoS One.

[9] Leeper HE, Caron AA, Decker PA, Jenkins RB, Lachance DH, Giannini C (2015). IDH mutation, 1p19q codeletion and ATRX loss in WHO grade II gliomas. Oncotarget.

[10] Eckel-Passow JE, Lachance DH, Molinaro AM, Walsh KM, Decker PA, Sicotte H, Pekmezci M, Rice T, Kosel ML, Smirnov IV, Sarkar G, Caron AA, Kollmeyer TM (2015). Glioma groups based on 1p/19q, IDH, and TERT promoter mutations in tumors. N Eng J Med.

[11] Yang P, Cai J, Yan W, Zhang W, Wang Y, Chen B, Li G, Li S, Wu C, Yao K, Li W, Peng X, You K (2016). Classification based on mutations of TERT promoter and IDH characterizes subtypes in grade II/III gliomas. Neuro Oncol.

[12] Louis DN, Perry A, Reifenberger G, von Deimling A, Figarella-Branger D, Cavenee WK, Ohgaki H, Wiestler OD, Kleihues P, Ellison DW (2016). The 2016 World Health Organization classification of tumors of the central nervous system: a summary. Acta Neuropathol.

[13] Bai H, Harmanci AS, Erson-Omay EZ, Li J, Coşkun S, Simons M, Krischek B, Özduma K, Omay SB, Sorensen EA, Turcan  Ş, Bakırcıǧlu M, Carrión-Grant G (2016). Integrated genomic characterization of IDH-1 mutant glioma malignant progression. Nat Genet.

[14] Hong CS, Hsieh JK, Edwards NA, Ray-Chaudhury A, Zaghloul KA (2016). IDH mutations may not preclude distant, trans-tentorial spreads in gliomas: a case report and review of the literature. World J Surg Oncol.

[15] Kitange G, Misra A, Law M, Passe S, Kollmeyer TM, Maurer M, Ballman K, Feuerstein BG, Jenkins RB (2005). Chromosomal imbalances detected by array comparative genomic hybridization in human oligodendrogliomas and mixed oligoastrocytomas. Genes Chromosomes Cancer.

[16] Faria MH, Khayat AS, Burbano RR, Rabenhorst SH (2008). c-MYC amplification and expression in astrocytic tumors. Acta Neuropathol.

[17] Jenkins RB, Xiao Y, Sicotte H, Decker PA, Kollmeyer TM, Hansen HM, Kosel ML, Zheng S, Walsh KM, Rice T, Bracci P, McCoy LS, Smirnov I (2012). A low-frequency variant at 8q24.21 is strongly associated with risk of oligodendroglial tumors and astrocytomas with IDH1 and IDH2. Nat Genet.

[18] Oktay Y, Ülgen E, Can Ö, Akyerli CB, Yüksel Ş, Erdemgil Y, Durası IM, Henegariu OI, Nanni EP, Selevsek N, Grossmann J, Erson-Omay EZ, Bai H (2016). IDH-mutant glioma specific association of re55705857 located at 8q24.21 involves MYC deregulation. Sci Rep.

[19] Ling H, Spizzo R, Atlasi Y, Nicoloso M, Shimizu M, Redis RS, Nishida N, Gafă R, Song J, Guo Z, Ivan C, Barbarotto E, De Vries I (2013). CCAT2, a novel noncoding RNA mapping to 8q24, underlies metastatic progression and chromosomal instability in colon cancer. Genome Res.

[20] Redis RS, Sieuwerts AM, Look MP, Tudoran O, Ivan C, Spizzo R, Zhang X, de Weerd V, Shimizu M, Ling H, Buiga R, Pop V, Irimie A (2013). CCAT2, a novel noncoding RNA in breast cancer: expression study and clinical correlations. Oncotarget.

[21] Tseng YY, Moriarity BS, Gong W, Akiyama R, Tiwari A, Kawakami H, Ronning P, Reuland B, Guenther K, Beadnell TC, Essig J, Otto GM, O’Sullivan MG (2014). PVT1 dependence in cancer with MYC copy-number increase. Nature.

[22] Sarver AL, Murray CD, Temiz NA, Tseng YY, Bagchi A (2016). MYC and PVT1 synergize to regulate RSPO1 levels in breast cancer. Cell Cycle.

[23] Okura H, Golbourn BJ, Shahzad U, Agnihorti S, Sabha N, Krieger JR, Figueiredo CA, Chalil A, Landon-Brace N, Riemenschneider A, Arai H, Smith CA, Xu S (2016). A role for activated Cdc42 in glioblastoma multiforme invasion. Oncotarget.

[24] Shonka NA, Theeler B, Cahill D, Yung A, Smith L, Lei X, Gilbert MR (2013). Outcomes for patients with anaplastic astrocytoma treated with chemoradiation, radiation therapy alone or radiation therapy followed by chemotherapy: a retrospective review within the era of temozolomide. J Neurooncol.

[25] Hirose Y, Sasaki H, Abe M, Hattori N, Adachi K, Nishiyama Y, Nagahisa S, Hayashi T, Hasegawa M, Yoshida K (2013). Subgrouping of gliomas on the basis of genetic profiles. Brain Tumor Pathol.

[26] Nakae S, Murayama K, Sasaki H, Kumon M, Nishiyama Y, Ohba S, Adachi K, Nagahisa S, Hayashi T, Inamasu J, Abe M, Hasegawa M, Hirose Y (2017). Prediction of genetic subgroups in adult supra tentorial gliomas by pre- and intraoperative parameter. J Neurooncol.

[27] Walker DR, Bond JP, Tarone RE, Harris CC, Makalowski W, Boguski MS, Greenblatt MS (1999). Evolutionary conservation and somatic mutation hotspot maps of p53: correlation with p53 protein structural and functional features. Oncogene.

[28] Leroy B, Anderson M, Soussi T (2014). TP53 mutations in human cancer: Database reassessment and prospects for the next decade. Hum Mutat.

[29] Holstege H, Joosse SA, van Oostrom CT, Nederlof PM, de Vries A, Jonkers J (2009). High incidence of protein-truncating TP53 mutations in BRCA1-related breast cancer. Cancer Res.

[30] Watanebe T, Nobusawa S, Kleihues P, Ohgaki H (2009). IDH1 mutations are early events in the development of astrocytomas and oligodendrogliomas. Am J Pathol.

[31] Koga Y, Yasunaga M, Moriya Y, Akasu T, Fujita S, Yamamoto S, Baba H, Matsumura Y (2009). Detection of the DNA point mutation of colorectal cancer cells isolated from feces stored under different conditions. Jpn J Clin Oncol.

[32] Solis OE, Mehta RI, Lai A, Mehta RI, Farchoukh LO, Green RM, Cheng JC, Natarajan S, Vinters HV, Cloughesy T, Yong WH (2011). Rosette-forming glioneuronal tumor: a pineal region case with IDH1 and IDH2 mutation analyses and literature review of 43 cases. J Neurooncol.

[33] Hirose Y, Sasaki H, Miwa T, Ohba S, Ikeda E, Abe M, Ikeda S, Kobayashi M, Kawase T, Hasegawa M, Yoshida K (2011). Whole genome analysis from microdissected tissue revealed adult supratentorial grade II-III gliomas are divided into clinically relevant subgroups by genetic profile. Neurosurgery.

